# Learning from Knowledge Graphs: Neural Fine-Grained Entity Typing with Copy-Generation Networks

**DOI:** 10.3390/e24070964

**Published:** 2022-07-11

**Authors:** Zongjian Yu, Anxiang Zhang, Huali Feng, Huaming Du, Shaopeng Wei, Yu Zhao

**Affiliations:** 1 Financial Intelligence and Financial Engineering Key Laboratory of Sichuan Province, Southwestern University of Finance and Economics, Chengdu 611130, China; yuzj@swufe.edu.cn (Z.Y.); fenghuali0723@163.com (H.F.); dhmfcc@smail.swufe.edu.cn (H.D.); 2191201z5003@smail.swufe.edu.cn (S.W.); 2 School of Computer Science, Carnegie Mellon University, Pittsburgh, PA 15213, USA; adamzhang1679@gmail.com

**Keywords:** knowledge graphs, fine-grained entity typing, copy-generation networks, cross-entropy

## Abstract

Fine-grained entity typing (FET) aims to identify the semantic type of an entity in a plain text, which is a significant task for downstream natural language processing applications. However, most existing methods neglect rich known typing information about these entities in knowledge graphs. To address this issue, we take advantage of knowledge graphs to improve fine-grained entity typing through the use of a copy mechanism. Specifically, we propose a novel deep neural model called CopyFet for FET via a copy-generation mechanism. CopyFet can integrate two operations: (**i**) the regular way of making type inference from the whole type set in the generation model; (**ii**) the new copy mechanism which can identify the semantic type of a mention with reference to the type-copying vocabulary from a knowledge graph in the copy model. Despite its simplicity, this mechanism proves to be powerful since extensive experiments show that CopyFet outperforms state-of-the-art methods in FET on two benchmark datasets (FIGER (GOLD) and BBN). For example, CopyFet achieves the new state-of-the-art score of 76.4% and 83.6% on the accuracy metric in FIGER (GOLD) and BBN, respectively.

## 1. Introduction

Fine-grained entity typing (FET) aims to infer the possible semantic type of an entity mention (i.e., a sequence of token spans representing an entity) [1,2]. Different from a traditional entity-typing task that typically classifies entities into coarse-grained types (e.g., *person, location, organization*) [3,4], FET aims to assign an entity with more specific types [5,6], which usually follow a hierarchical structure that can provide more semantic information about the entity [7,8], such as */person/politician*, */book/author*, etc. FET is a significant subtask of named-entity recognition (NER) [9] for downstream natural language processing (NLP) applications, such as relation extraction [10,11], question answering [12,13], knowledge base population [14], and recommendation [15,16].

In FET, knowledge graphs (KGs) usually play an important role. For example, given large-scale KGs, FET systems resort to distant supervision [10] to generate large training corpora [9,17,18] (i.e., to label entity mentions in the training corpus with all types associated with the entity in KGs). Although distant supervision can eliminate the high cost in labeling training data with KGs, how to efficiently encode a KG’s typing knowledge into FET model is still underexplored.

In this paper, we concentrate on how to take advantage of KGs to improve FET in the process of type inference. In fact, the correct type information about a mention usually can be found in large-scale knowledge graphs, such as Freebase [19], YAGO [20], DBpedia [21], OntoNotes [22], and Few-nerd [23], which typically have already recorded a large number of entity-typing facts with various context from corpus. Consider the following example from Wikipedia: “In 2006, *Obama* released *The Audacity of Hope* that expanded upon the themes in their convention speech.” The entity mention “*The Audacity of Hope*” can directly be predicted as a *book* by copying the type information of the known entity-typing fact (“*The Audacity of Hope*”, */things/book*) from KGs to the mention. For cases that require the understanding of the entity context, using a copy mechanism is also quite beneficial. In the previous example where *“Obama”* is the entity mention, copying all the types of *“Barack Obama”* in KGs (i.e., */people/person*, */book/author*, */person/politician*, etc.) as the priority typing candidates of the mention is still informative for inferring the correct type (i.e., */book/author*) that fits the context best, since they can substantially narrow down the optimal list of possible type labels (referred to as the type’s *copying vocabulary* from here onward). According to the statistics in Table 1, over 49% and 64% of all the manually annotated mentions’ typing facts in FIGER (GOLD) [9] and BBN [24] (testing data) have already been included in their existing KGs, respectively, which inspires us to improve FET by learning rich known entity-typing information from KGs.

To this end, we incorporate a copy mechanism in fine-grained entity typing, and propose a novel deep neural model called CopyFet for FET via a copy-generation mechanism. A copy mechanism can significantly avoid unnecessary mistakes and improve the accuracy in the type inference process. It is similar to the copy mechanism in the keyphrase generation [25] and abstractive summarization [26,27], which allows a language generator to copy items from the source text directly, in order to help generate reliable results that keep salient information from the source text.

**Figure 1:**
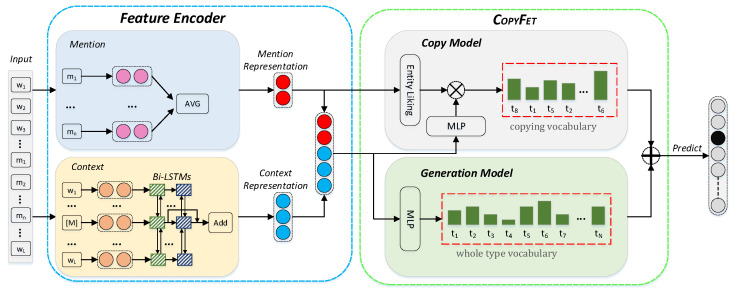
The overall framework of our proposed model CopyFet. In Feature Encoder (left box), it obtains the *mention representation* by averaging (Equation (2)) and the *context representation* by Bi-LSTM (Equation (3)). In CopyFet (right box), the green bar indicates the probability score calculated by the **copy model** (Section 3.1) and the **generation model** (Section 3.2). The copy model is able to learn to predict from a much more delimited candidate space, i.e., the type’s *copying vocabulary*, than the whole type vocabulary, on which the generation model makes a prediction. The final type prediction agrees with both of them (Section 3.3).

Specifically, CopyFet includes two submodels of inference, i.e., a copy model and a generation model, as shown in Figure 1. CopyFet has two submodels: **(i)** the first one conducts the regular way of making type inference from the whole type set, i.e., generation model; **(ii)** the second one uses the new copy mechanism which can identify the semantic type of a mention with reference to the type’s *copying vocabulary* from KGs, called copy model. Both models are combined to build the final type inference model. Since it is unknown that a certain entity mention corresponds to a certain entity in KGs, we propose to perform entity linking as a solution to generate the type’s *copying vocabulary*.

Extensive experimental results on two benchmark FET datasets demonstrate that the proposed CopyFet can effectively conduct fine-grained entity typing by incorporating a copy model with a generation model in both training and inference. For example, CopyFet achieves 76.4% and 83.6% absolute strict accuracy on the benchmark datasets FIGER (GOLD) and BBN, respectively.

The contributions of this paper are threefold: (1) We propose to take advantage of knowledge graphs to improve fine-grained entity typing through the use of a copy mechanism. (2) Specifically, we propose CopyFet, a simple but effective neural fine-grained entity-typing model that incorporates a copy mechanism in FET via a copy-generation framework. (3) We conduct empirical experiments on two benchmark datasets, which demonstrate that incorporating a copy mechanism highlights the superiority of the proposed CopyFet over previous SOTA models in a FET task.

The structure of the paper is as follows. In Section 2, we provide a brief review of related works. In Section 3, we describe the methodology of our model. In Section 4, we present the cross-entropy learning method. Section 5 presents experimental results followed by their discussion. Finally, Section 6 gives the conclusion and future directions of this research.

## 2. Related Work

To make this paper self-contained, we introduce some related topics here on fine-grained entity typing, and copy mechanism.

### 2.1. Fine-Grained Entity Typing

The FET task was first introduced by [9,28]. Different from lexicon-level [29], discourse-level [30], and corpus-level [31,32] FET, most previous works consider sentence-level entity typing. The progress of FET has primarily focused on the following directions.

*Neural Network Model.* Different from early heuristic hand-crafted feature-based models [9,28,33] and embedding-based methods [1], the neural models are expected to learn better latent representations for mention and context [8,34,35]. For instance, ref. [36] firstly used recurrent neural networks (RNNs) to recursively obtain a vector representation of each entity mention. Refs. [37,38] proposed to incorporate an attention mechanism with LSTM. Ref. [39] proposed a CNN-based FET model. Refs. [40,41] proposed attentive neural models that also encoded latent type representations besides mention and context. Refs. [8,41] found that the pretrained language model ELMo [42] performed better than BERT [43] as the input of a neural model. Our model builds upon these progress and takes advantage of the state-of-the-art neural network architecture.

*Incorporating Knowledge Graphs.* Different from distant supervision that only takes advantage of KGs to build training data, a few researchers focused on incorporating KGs into FET models. Ref. [37] proposed to improve FET with knowledge attention which learns the relational information from KGs. Ref. [34] proposed to enrich the mention features by adding a KG-type representation obtained from KGs. However, they did not directly utilize the entity-typing facts in KGs for improving FET.

*Denoise.* Most typical FET datasets, such as FIGER (GOLD) [9], BBN [24], and OntoNotes [18,28], are labeled with KGs by distant supervision [10], which inevitably bring noise to training data. Several studies aimed to address these problems by heterogeneous partial label embedding [17,44,45], hierarchy-aware loss normalization [38], language model enhancement [46], filtering function [47], compact latent space clustering [48], virtual adversarial learning [49], attentive graph convolution network [50], and automatic relabeling [51]. There would be a concern about the noise issue for our model; however, it is not the main focus of this paper. We believe that our model could be further boosted by adding a denoising module, which we reserve for future.

*Others.* There are some other points concerned in FET. Recently, some researchers have focused on FET in KGs, also known as knowledge graph entity typing (KGET), which is a subtask of knowledge graph completion [52], by using external data outside KGs [53,54], or only with structural relational information in KGs [52,55]. Some have concentrated on encoding the hierarchical characteristics of fine-grained type in their models [7,44] and on zero-shot entity typing [56,57].

### 2.2. Copy Mechanism

The copy mechanism is widely used in various natural language generation tasks, such as sequence-to-sequence learning [58,59], keyphrase generation [25], abstractive summarization [27], and entity prediction [60]. Ref. [58] proposed the pointer networks, which used attention as a pointer that could select a member of an output sequence directly from the input, which could be seen as a copy model with an attention mechanism. However, it could not make prediction using external lexemes besides the input sequence. Ref. [59] proposed CopyNet to solve this issue in a hybrid end-to-end way, which incorporated the copy mechanism with a generation model which yielded external lexemes that did not appear in the input sequence. Based on [25,59] proposed to incorporate a copy mechanism with a recurrent neural network (RNN)-based generation model for deep keyphrase generation. To enhance the copy mechanism, ref. [61] proposed SeqCopyNet, which not only could copy single words but also copy subsequences from the input text. Ref. [27] proposed a transformer model with copy mechanism for abstractive summarization. Inspired by previous works, we utilize the copy mechanism with the typing characteristics of knowledge graphs. To the best of our knowledge, we are the first to attempt to incorporate the copy mechanism in neural fine-grained entity typing.

## 3. Methodology

In this section, we introduce the details of the proposed model CopyFet. We first give the notations and then introduce the feature encoder and the model architecture, which includes a copy model and a generation model.

***Notations.*** Given an entity mention *m* and its context *c* in a sentence *s*, and a set of type tags T, our model aims to predict the probability of each type t∈T for this mention. We denote $w_{1},w_{2},\cdots ,\left[m_{1},m_{2},\cdots ,m_{n}\right],\cdots ,w_{L}$ as the words in the sentence, where $m_{1},m_{2},\cdots ,m_{n}$ denotes the words in entity mention. *L* and *n* denote the number of words in the sentence *s* and mention *m*. Boldfaced m and c represent the embedding vector of mention *m* and context *c*, respectively. Besides containing lots of entity facts, a knowledge graph G also provides a large amount of existing entity-typing instances, i.e., $G=\{\left(e,t\right)|e\in E,t\in \hat{T}\}$. E and $\hat{T}$ represent the set of entities and types, respectively. For each entity mention *m* and its corresponding entity *e*, we build a bounded subset of T which is specific to *m* (namely a type’s *copying vocabulary* for *m*) as $T_{m}$. It consists of all the types that have been labeled as the types in known entity-typing facts with entity *e* in the KG.

Since the labels in type tag set T and KG type set $\hat{T}$ may not be exactly the same, it needs type-mapping processing. The *copying vocabulary* $T_{m}$ is an *N*-dimensional few-hot indicative vector. *N* stands for the size of T. The value of types in the *copying vocabulary* are marked 1 while others are set to 0. The fine-grained entity typing task is to infer the type probability distribution in type tags space T given the mention *m* and context *c*, i.e., $\begin{array}{c} p(t|m,c)=? \end{array}$.

***Feature Encoder.*** We concatenated the entity mention representation m and its context representation c in the sentence as the feature vector x, as follows:

(1) $$x=\left[\begin{array}{c} m\\ c \end{array}\right]$$

***Entity Mention Representation.*** The entity mention representation m was simply calculated by averaging the embeddings of all words in entity mention [$m_{1},m_{2},\cdots ,m_{n}$] [37], as follows:

(2) $$m=\frac{1}{n}\sum_{i=1}^{n}m_{i}\;,$$

where *n* represents the length of the entity mention.

***Context Representation.*** We used a bidirectional LSTM (BiLSTM) to encode context representation. We first utilized a special token to denote the mention, as the token “[M]” in Figure 1. Then, the word embeddings of the modified context words $\left\{w_{1},w_{2},\cdots ,\left[M\right],\cdots ,w_{L}\right\}$ were fed into two layers of BiLSTM (bidirectional long short-term memory), and the context representation c was the sum of the BiLSTM layers’ outputs:

(3) $$c=h_{m}^{1}+h_{m}^{2}\;,$$

where $h_{m}^{1}$ and $h_{m}^{2}$ are the output of the first and the second layer of BiLSTM for [M], respectively.

### 3.1. Copy Model

The copy model was designed to identify types from its corresponding *copying vocabulary* that stemmed from the known entity-typing instances in existing KGs.

We first dealt with the training dataset to build the type’s *copying vocabulary* for each mention, i.e., $T_{m}$, which contained three steps: (1) Given a mention *m*, we utilized a simple entity-linking (EL) algorithm (similar to [34]) to retrieve its corresponding entity in the KG. Specifically, we directly linked the mention to the entity with the largest commonness score [62], which indicated the probability of an entity given the entity mention. The commonness score was calculated based on the anchor links in Wikipedia. (2) If the EL algorithm returned an entity, we obtained the types of this entity from the KG. (3) Since the types in the KG may be different from the target type set T, following the rules used in [56], we mapped them to the type tags in T. $T_{m}$ was an N-dimensional multi-hot vector and the value of types in the type’s *copying vocabulary* was 1, while others were 0. Note that if the result of EL is NULL, we simply set $T_{m}$ as a zero vector.

If the mention *m* has the type’s *copying vocabulary*$T_{m}$, CopyFet increases the probability value calculated for the candidate types that are chosen from the *copying vocabulary*. Specifically, the copy model first builds an indicator vector $v_{T}$ with a multilayer perceptron (MLP):

(4) $$v_{T}=f_{c}\left(x\right)\;,$$

where $f_{c}$ is a three-layer MLP with Relu activation, and the vector $v_{T}$ is an N-dimensional indicator vector. *N* is the size of the type’s tag vocabulary T.

To decrease the probability value of some types that do not belong to the type’s copying vocabulary (i.e., uninterested types for the copy model), CopyFet implements an element-wise multiplication between the index vector $v_{T}$ and the indicator vector $T_{m}$. Formally, the type prediction distribution of the copy model is defined as follows:

(5) $$p_{c}\left(t|m,c\right)=v_{T}⊙T_{m}\;,$$

where $\begin{array}{c} p_{c}\left(t|m,c\right) \end{array}$ stands for the prediction probabilities on the type’s *copying vocabulary*. The maximum item of $p_{c}\left(t|m,c\right)$ indicates the type will be copied from the type’s *copying vocabulary*. The basic idea behind the copy model is that it is more beneficial to learn to predict from a small candidate set than the whole type vocabulary. However, entity-typing facts may be out-of-KG. Thus, it needs an additional generation model to infer such typing facts.

### 3.2. Generation Model

With the same mention *m* and context *c*, the generation model concentrates on type inference by selecting the type from the whole type set T. The inference made by the generation model treats the typing fact as a new one without any mentions to the knowledge graph. Similar to the copy method, the generation model also builds a whole type vocabulary query vector $p_{g}$ as follows:

(6) $$p_{g}\left(t|m,c\right)=f_{g}\left(x\right)\;,$$

where $f_{g}$ is a three-layer MLP with Relu activation. Similar to $p_{c}\left(t|m,c\right)$ in the copy model, $\begin{array}{c} p_{g}\left(t|m,c\right) \end{array}$ stands for the predicted probability distribution among the whole type set. The largest score in $p_{g}\left(t|m,c\right)$ denotes the corresponding type we inferred in the whole type set from the generation model.

### 3.3. Incorporating Copy Model with Generation Model for FET

To make type distribution prediction regarding a query p(t|m,c)=?, both copy model and generation model predict a type among their candidate type sets. As shown in Figure 1, CopyFet incorporates the predicted results from both models as follows:

(7) $$\begin{array}{c} \begin{array}{c} p\left(t|m,c\right)=\lambda *p_{c}\left(t|m,c\right)+\left(1-\lambda \right)*p_{g}\left(t|m,c\right)\;, \end{array} \end{array}$$

where $p_{c}\left(t|\cdot \right)$ stands for the copy model, and $p_{g}\left(t|\cdot \right)$ stands for the generation model. λ is a hyperparameter for the trade-off between copy model and generation model.

To cope with the *overly specific* issue that usually biases the model towards popular subtypes instead of generic ones, i.e., preferring *politician* over *person*, we designed a recursive selecting method. For each stage, we chose the type that had the maximum combined probability in that depth and then we went deeper. We implemented it recursively until the maximum probability was smaller than a threshold. For the sake of simplicity, let us suppose the maximum depth of the type hierarchy is 2 without loss of generality. Formally, we denote $T^{1}$ as the type set of first level, and $T_{t_{i}}^{2}$ stands for the children type set (second level) of a specific type $t_{i}^{1}\in T^{1}$. The final output fine-grained entity type prediction $\hat{t}$ changes with a threshold β as follows:

(8) $$\begin{array}{cc} t_{i}^{1} & =\underset{t\in T^{1}}{\arg\max}p\left(t|m,c\right)\;,\\ t_{j}^{2} & =\underset{t\in T_{t_{i}}^{2}}{\arg\max}p\left(t|m,c\right)\;,\\ \hat{t} & =p\left(t=t_{j}^{2}|m,c\right)>\beta \;?\;t_{j}^{2}\;:\;t_{i}^{1}\;, \end{array}$$

where $t_{i}^{1}$ indicates the output type with the highest probability in the first level, and $t_{j}^{2}$ indicates its subtype with the highest probability in $T_{t_{i}}^{2}$. Here, β∈(0,1) is a hyperparameter acting as a threshold that controls the specific degree of the hierarchical type. The higher β is, the more coarse-grained the final output type should be.

## 4. Cross-Entropy Loss Function for Optimization

Since in the training set, there were multiple ground truths for one mention, the objective function was defined as the element-wise cross-entropy over all entity mentions:

(9) $$L=-\sum_{i}t_{i}^{*}\log p\left(t\right)+\left(1-t_{i}^{*}\right)\log\left(1-p\left(t\right)\right)\;,$$

where $t^{*}$ indicates the ground truth types of the mention. Since the training data were automatically generated by linking the mention to all labels in the KG, which was the same as the copy vocabulary, it may cause the model to overfit the weakly labeled training data. This is fine for most types of entities such as */locations* and */organizations* since they usually have the same type in different context; however, this is problematic for other context-dependent entities, such as */person* entity mentions. To alleviate overfitting issue, we added a random fine-grained type label that did not belong to this entity when building the type copying vocabulary. During training, we employed dropout in two LSTM layers and MLP layers.

## 5. Experiments

In this section, we evaluate the effectiveness of the proposed CopyFet with two public datasets.

### 5.1. Datasets

We used two publicly available benchmark datasets for FET experiments, including Wiki/FIGER (GOLD) [9] and BBN [24]. The statistics of the percentage of the entity mentions’ typing facts of the two benchmark datasets (i.e., testing data) that have been already covered by existing KGs are shown in Table 1, and other statistics are included in Table 2.

***Wiki/FIGER (GOLD)***. Ref. [9] extracted a dataset from Wikipdia articles and news reports to form the training, validation set, and testing data, and annotated entity mentions using 113 types with a two-level hierarchy.

***BBN*** [24] is based on a portion of the one million word Penn Treebank corpus from Wall Street Journal articles and is completely manually annotated using 56 types with a two-level hierarchy. Ref. [44] regenerated the training corpus via distant supervision.

### 5.2. Baselines

To demonstrate the effectiveness of our proposed model CopyFet, we compared results with several state-of-the-art FET models:

- AFET [44]: one of the most widely used FET model. AFET models the samples with only one label and samples with multiple labels separately with a partial label loss to handle noisy labels.
- Attentive [63]: a popular attention-based neural network model which uses attention mechanism to focus on relevant information.
- AAA [45]: an extension of AFET which jointly encodes entity mentions and their context representation.
- NFETC [38]: a very popular model which formulates FET as a single-label classification problem with hierarchy-aware loss.
- NFETC-CLSC [48]: an influential extension of NFETC which utilizes imperfect annotation as model regularization via compact latent space clustering to address the confirmation bias problem.
- IFETET [34]: a FET model which utilizes entity type information from a KB obtained through entity linking to form the final feature vector of a mention.
- NDP [7]: a random-walk-based model which weighs out noise with a loss function.
- HFET [41]: a popular ELMo-based pretrained language model which adopts a hybrid type classifier.
- HET [8]: a recent model that takes the hierarchical ontology into account with a multilevel learning-to-rank loss and gains great performance improvement.
- FGET-RR [50]: a recent model that refines the noisy mention representations by attending to corpus-level contextual clues prior to the end classification.
- Box [64]: a recent box-based model for fine-grained entity typing.

### 5.3. Experimental Settings

In CopyFet, we evaluated the performance by the strict accuracy (Strict Acc), loose macro F-score (Macro-F1), and loose micro F-score (Micro-F1), which are the most widely used evaluation settings for FET systems [9]. We used pretrained word embeddings from [65]. The settings are shown in Table 3. For training our model, we selected the parameters λ and β ∈ {0.1, 0.2, 0.5, 0.7} based on the validation set. Finally, the λ was set to 0.5 and 0.7 on Wiki/FIGER (GOLD) and BBN, respectively. The β was set to 0.5 on both Wiki/FIGER (GOLD) and BBN.

### 5.4. Results and Analysis

Table 4 demonstrates the results of fine-grained entity typing. We can observe that our CopyFet outperforms all baselines for fine-grained entity typing in terms of all metrics on Wiki/FIGER and BBN. Specifically, our model improves the strict accuracy on the two datasets with values of 76.4 and 83.6, respectively, which confirms the capability of CopyFet to incorporate the copy mechanism for FET using the copy-generation mechanism and to infer types for mentions in text. These results are in line with our intuition, which indicated that the proposed model was capable of leveraging entity-typing information from existing KGs to build the type’s *copying vocabulary*. It is the main feature that leads to the better performance of CopyFet. It can substantially narrow down the optimal list of possible type labels for unlabeled mentions, and thus can significantly improve the performance of FET.

### 5.5. Ablation Study

**Figure 2:**
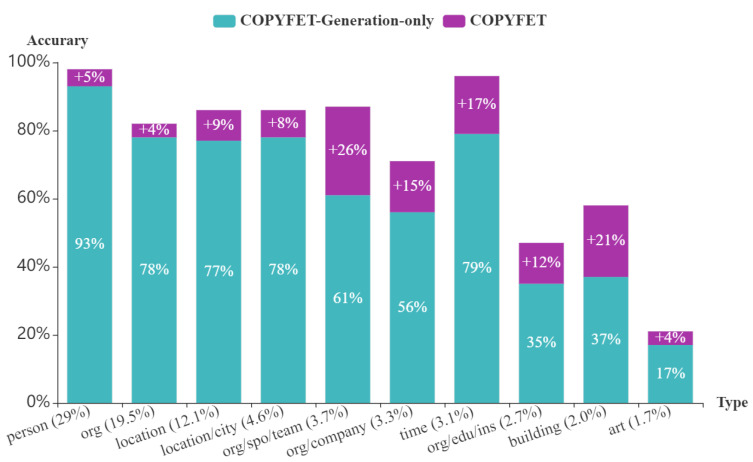
Performance analysis of CopyFet on the top 10 types present in FIGER dataset. In these ten types, CopyFet performs better than CopyFet-Generation-only.

To evaluate the different component of our model CopyFet, we conducted an ablation study. To this end, we generated a variant of CopyFet by deleting the use of its copy components (called CopyFet-Generation-only), and compared the fine-grained entity-typing performance on Wiki/FIGER and BBN. Table 5 shows the fine-grained entity-typing results by the variant of our model CopyFet. We can observe that the copy model is significant. Deleting the copy module leads to drops of all metrics on strict accuracy in Wiki/FIGER and BBN, respectively, which indicates that learning to infer types for unlabeled mentions by referring to the known entity-typing facts in KGs can be quite beneficial. The results demonstrate our model CopyFet can successfully take into account the known entity-typing information from existing KGs via the copy mechanism to improve FET. Next, we analyzed the detailed results of CopyFet compared to CopyFet-Generation-only. In Figure 2, we present the type-wise performance for the top-10 most frequent types in the FIGER testing dataset. Compared to CopyFet-Generation-only, CopyFet performs better in all types.

### 5.6. Case Study

Table 6 gives two examples of fine-grained entity-typing results on the FIGER and BBN testing set. For example, given a mention of “*Unitec Institute of Technology*” and its context, the possible fine-grained types are inferred by CopyFet-Generation-only and CopyFet, respectively. The former makes a false prediction (i.e., /location), while the latter can make a positive inference since the known entity-typing fact (“*UNITEC*”, /organization/educational_institution) has already been included in the KG (e.g., Freebase). This is quite helpful for the prediction, which illustrates the efficacy of the copy model in CopyFet.

## 6. Conclusions and Future Work

In this paper, we proposed a novel model architecture for fine-grained entity typing using KGs. The proposed model leveraged the popular copying mechanism that “copies” an inferred fine-grained type probability distribution of the target from a knowledge graph. The copied distribution was then combined with the output of a regular generation model that predicted the distribution of the full set of types. This new method achieved new SOTA results on FET, outperforming previous methods based on weak supervision or knowledge injection using KGs. The ablation analysis showed that the copying module of the model contributed significantly to the improved prediction quality. Interesting future work directions include exploring how to make the construct operation of mapping from the target types to the KG types in this model soft (currently it is one-hot) and trainable and adding a denoising module in the copy-generation networks.

## Figures and Tables

**Table 1:** The percentage of the entity mentions’ typing facts that have been included in the existing knowledge graphs. The statistical analysis indicates they are over **49%** and **64%** in two benchmark datasets FIGER (GOLD) and BBN (testing data), respectively. These observations are the key motivation that inspired us to take advantage of knowledge graphs to improve fine-grained entity typing through the use of a **copy mechanism** in this paper.

Benchmark FET Datasets	# Type	# Testing Mentions	# Typing Facts Included in KG	KG Coverage
Wiki/FIGER (GOLD) [9]	128	563	280	**49.73%**
BBN [24]	56	13,282	8505	**64.03%**

**Table 2:** The statistics of the two benchmark datasets.

Dataset	# Train	# Dev	# Test	# Label	Depth
FIGER [9]	4,932,761	2000	563	128	2
BBN [24]	4,695,789	2000	13,282	56	2

**Table 3:** Hyperparameter settings for training on two datasets.

Parameter	Wiki/FIGER (GOLD)	BBN
Learning rate	1 × 10^−3^	1 × 10^−3^
Batch size	256	256
Word vector size	300	300
LSTM hidden	250	250
dropout	0.5	0.5
λ	0.5	0.7
β	0.5	0.5

**Table 4:** **Fine-grained entity typing results.** Evaluation of different models on Wiki/FIGER and BBN. The results of baselines are directly taken from the original papers. The best scores are in **bold**.

Model	Wiki/FIGER (GOLD)			BBN		
	Strict Acc.	Macro F1	Micro F1	Strict Acc.	Macro F1	Micro F1
AFET [44]	53.3	69.3	66.4	67.0	72.7	73.5
Attentive [63]	59.7	80.0	75.4	48.4	73.2	72.4
AAA [45]	65.8	81.2	77.4	73.3	79.1	79.2
NFETC [38]	68.9	81.9	79.0	72.1	77.1	77.5
NFETC-CLSC [48]	-	-	-	74.7	80.7	80.5
IFETET [34]	74.9	86.2	84.0	82.1	88.1	89.3
NDP [7]	67.7	81.8	78.0	72.7	76.4	77.7
HFET [41]	62.9	83.0	79.8	55.9	79.3	78.1
HET [8]	65.5	80.5	78.1	75.2	79.7	80.5
FGET-RR [50]	71.0	84.7	80.5	70.3	81.9	82.3
Box [64]	-	79.4	75.0	-	78.7	78.0
CopyFet (Ours)	**76.4**	**86.7**	**84.6**	**83.6**	**89.4**	**89.9**

**Table 5:** **Ablation study**. Fine-grained entity typing results by different variants of our model CopyFet on Wiki/FIGER and BBN.

Model	Wiki/FIGER (GOLD)			BBN		
	Strict Acc.	Macro F1	Micro F1	Strict Acc.	Macro F1	Micro F1
CopyFet-Generation-only	69.9	82.7	80.6	79.8	86.8	87.9
CopyFet	**76.4**	**86.7**	**84.6**	**83.6**	**89.4**	**89.9**

**Table 6:** Example type predictions on FIGER and BBN testing sets using CopyFet-Generation-only and CopyFet. **Bold** indicates the true prediction.

Data	Mention and Context	Known Facts in KGs	CopyFet-Generation-only	CopyFet
Wiki	The study is from the *Unitec Institute of Technology*, Auckland, New Zealand.	(*UNITEC*, /organization) (*UNITEC*, /organ./edu-cational_inst.)	/location	**organization/edu-cational_institution**
BBN	The Fleet Street reaction was captured in the Guardian headline, “ *Departure Reveals Thatcher Poison*.”	(*D. R. T. P.*, /art) (*D. R. T. P.*, /work_of_art)	/organization	**/work_of_art**
